# A fourth subtype of retinoic acid receptor-related orphan receptors is activated by oxidized all-*trans* retinoic acid in medaka (*Oryzias latipes*)

**DOI:** 10.1186/s40851-017-0074-7

**Published:** 2017-08-11

**Authors:** Kotowa Sakai, Haruka Fukushima, Yuya Yamamoto, Toshitaka Ikeuchi

**Affiliations:** 1 Nagahama Institute of Bio-Science and Technology, Graduate School of Biosciences, 1266, Tamura, Nagahama, Shiga 526-0829 Japan; 2 Department of Bioscience, Nagahama Institute of Bio-Science and Technology, Faculty of Bioscience, Nagahama, Shiga 526-0829 Japan; 3 Department of Bioscience, Nagahama Institute of Bio-Science and Technology, Laboratory of Molecular Response to Environmental Signal, Faculty of Bioscience, Tamura-cho 1266, Nagahama, Shiga 526-0829 Japan

**Keywords:** ROR, ATRA, Medaka, Evolution

## Abstract

**Background:**

The three known subtypes of the retinoic acid receptor-related orphan receptor (ROR) have been implicated in the control of immunity, brain function, and circadian rhythm in mammals. Here, we demonstrate by phylogenetic analysis that there were originally four subtypes of RORs in vertebrates. One of the novel *ror* paralogs, *rord1* (*rorca* in the Ensembl database), is conserved among teleosts, but absent in mammals. Using medaka (*Oryzias latipes*) as a model teleost, we evaluated the expression pattern of this gene, its transactivational properties for endogenic chemicals, and its ability to activate the promoters of putative target genes.

**Results:**

In eyes, the transcript of *rord1* was expressed at higher levels during the day than at night. Interestingly, cholesterol derivatives, which are well-known ligands for mammalian RORs, did not efficiently promote transcriptional activity via RORd1. Thus we sought to identify the ligands that regulate the transcriptional activity of RORd1 using a luciferase reporter cell-based screening system. Using this system, we identified two metabolites of all-*trans* retinoic acid (ATRA), 4OH-ATRA and 4-keto ATRA, as potential ligands of RORd1. Moreover, RORd1 activated the promoter of *cyp26a1* in a 4OH-ATRA -dependent manner.

**Conclusions:**

A novel *ror* subtype, rord has two paralogs, *rord1* and *rord2*, in teleost. Rord1 mRNA is highly abundant in the eyes of medaka during light periods, suggesting that *rord1* expression is involved in the regulation of circadian rhythm. We identified two ATRA metabolites, 4OH-ATRA and 4 K–ATRA, as endogenous candidate ligands of RORd1. We also show that 4-oxygenated ATRA metabolites have the potential to activate cyp26a1, the metabolic enzyme of ATRA. Our results support the notion that RORd1 is involved in the metabolism of ATRA in medaka.

**Electronic supplementary material:**

The online version of this article (doi:10.1186/s40851-017-0074-7) contains supplementary material, which is available to authorized users.

## Background

The retinoic acid receptor (RAR)-related orphan receptors alpha, beta and gamma (RORα-γ, NR1F1–3 or RORa-c) constitute a subfamily of nuclear receptors that appear to function as ligand-dependent transcription factors [1]. Thus, although RORs may function as constitutively active receptors, several endogenous substances have been identified as agonists; for example, melatonin [2], cholesterol [3] and cholesterol sulfate [4] can activate RORa, whereas 20α*-*hydroxycholesterol (20α-OHC), 22(R)-hydroxycholesterol, 25-hydroxycholesterol [5] and 7β, 27-dihydroxycholesterol [6] have the ability to activate RORc. All-*trans* retinoic acid (ATRA) was not reported as an agonist, but rather as an inverse agonist of RORb [7], despite the view that this receptor is a ‘retinoic acid’ receptor-related receptor.

The RORs have been implicated in the regulation of immunity, brain function, retinal development, glucose and lipid metabolism, and circadian rhythm [8]. They exert these effects by modulating gene transcription after binding as monomers to ROR response elements (ROREs) consisting of the consensus sequence WAWNTRGGTCA in the regulatory regions of target genes [9, 10]. As opposed to the breadth of detail obtained from mammals, very little is known about RORs in basal vertebrates. Interestingly, five subtypes of ROR—RORa, RORb, RORc, RORca, and RORcb—are found in the genome database of medaka, *Oryzias latipes*.

Many gene families in vertebrates were generated during two rounds of whole-genome duplication that occurred early in the evolution of Chordata [11–13]. An additional whole genome duplication event occurred in the fish lineage [14]. For example, four ROR paralogs are expected in humans, such as types a, b, c and d. In the case of teleost fish, there may be as many as eight paralogs, types a1, a2, b1, b2, c1, c2, d1, and d2. However, medaka clearly does not fit this scenario, highlighting the need for a review of ROR classification.

In the present study, we sought to categorize the ROR paralogs in vertebrates, and identified the novel ROR paralogs, RORd1 and RORd2. Subsequently, we investigated the time- and tissue-dependent expression profiles of these genes, and identified potential ligands for RORd1 as a representative subtype of the RORds. We also demonstrate RORd1-dependent regulation of the *bmal1a* and *cyp26a1* genes.

## Methods

### Phylogenetic analysis

ROR protein sequences were retrieved from the Ensembl database. Amino acid sequence IDs were as follows: human (*Homo sapiens*) RORa: BAH02285.1, RORb: BAH02286.1, RORg: BAH02287.1; mouse (*Mus musculus*) RORa2: NP_001276845.1, RORb2: ABG77270.1, RORg: AAC53501.1, chicken (*Gallus gallus*) RORa: NP_001276816.1, RORb: NP_990424.1, RORg: XP_015135499.1, RORblike: XP_003642912.2; sea turtle (*Chelonia mydas*) RORb: XP_007065615.1, RORblike: XP_007068624.1; soft-shelled turtle (*Pelodiscus sinensis*) RORa: XP_014435946.1, RORbX1: XP_006137881.1, RORg: XP_014427156.1, RORblike: XP_006129037.1; frog (*Xenopus tropicalis*) RORaX1: XP_012822203.1, RORb: XP_002940077.1, RORblike: XP_002938868.1; medaka (*Oryzias latipes*) RORaX1: XP_004069686.1, RORb: ENSORLT00000015579.1, RORgX1: XP_011483568.1, RORblike: XP_004067713.1, RORcb: ENSORLT00000018666.1, RARb: ENSORLT00000010680.1; Nile tilapia (*Oreochromis niloticus*) RORaX2: XP_005470779.1, RORbX1: XP_005473204.1, RORgX1: XP_013130314.1, RORblike: XP_005478800.1, RORblikeX2: XP_005476199.1; zebrafish (*Danio rerio*) RORa2: BAF76726.1, RORb: ABO15413.1, RORc: ENSDART00000149097.2, RORca: NP_001076288.1, RORcb: NP_001264023.1; grass carp (*Ctenopharyngodon idella*) RORg1: AFC34773.1, RORg2: AFC34774.1; fugu (*Takifugu rubripes*) RORaX2: XP_011605662.1, RORca: ENSTRUT00000007520.1, RORcb: ENSTRUT00000013484.1; spotted gar (*Lepisosteus oculatus*) RORaX2: XP_015199181.1, RORbX1: XP_006627046.2, RORblikeX1: XP_015221273.1. Amino acid sequences of RORs were aligned using ClustalW displayed in MEGA 7.0.14 (Molecular Evolutionary Genetics Analysis, ver. 7.0.14), and manually adjusted. Phylogenetic trees were constructed with MEGA 7.0.14 software using the neighbor-joining method [15] with branch support values estimated using bootstrap with 1000 replications.

### Experimental animals

All of the animal experiments described below were conduct in compliance with institutional guidelines and approved by the Animal Experiment Committee of Nagahama Institute of Bio-Science and Technology. Mature fish of the orange-red variety of medaka were acclimated to conditions of 24 °C on a 14-h light, 10-h dark cycle for at least 2 weeks prior to sampling. All fish were anaesthetized in aqueous solution of 0.01% ethyl *p*-aminobenzoate buffered with 0.01% sodium hydrogen carbonate before euthanasia. For expression analysis, brains and peripheral tissues from three fish from both sexes (body length = 22–31 mm) were collected every fourth hour [Zeitgeber time (ZT); 3, 7, 11, 15, 19, 23 h after “light on”]. Samples were immersed in Sepasol RNA I Super G (Nacalai Tesque) and kept at −80 °C until use.

### Quantitative Real-time (RT)-PCR

Total RNA from each tissue was extracted using Sepasol RNA I Super G (Nacalai Tesque) according to the manufacturer’s instruction. Total RNA was reverse-transcribed and genomic DNA was removed using ReverTra Ace qPCR RT Master Mix with gDNA Remover (TOYOBO). Quantitative Real-time (RT)-PCR was conducted with SsoAdvanced Universal SYBR Green Supermix (BIO-RAD) using the MiniOpticon real-time PCR system (BIO-RAD). The primer set for *rord1* (XM_004067665.2) was 5′-TGACGTCCAGAAGGTTCAAAAGT-3′ (forward) and 5′-TCTCCTCAGACGCTCCTTTATTCT-3′ (reverse). The primer set for *bmal1a* (scaffold212: 822,689–822,960) was 5′-CTCCTTCTACGAGTACTTCCATCAG-3′ (forward) and 5′-ATGGTGTTGGTAGAGACGATGTACT-3′ (reverse). The primer set for *cyp26a1* (ENSORLG00000014516) was 5′- CCCAGCACAGGACGAAGAA-3′ (forward) and 5′- CTGGATGACGGGGATGTAGAG-3′ (reverse). These primers were designed using Primer3 ver. 0.4.0 (1, 2). The conditions for PCR were as follows: initial denaturation at 98 °C for 2 min, 40 amplification cycles including denaturation at 98 °C for 2 s, annealing at 60 °C for 5 s. Measured values were normalized over the amount of total RNA and presented as copy/μg-total RNA.

### Development of medaka *ror1d* expression construct, transfection into HEK293 and ligand screening by luciferase assay

A chimera expression construct for Gal4-RORd1, which contains the cDNA of the hinge domain and the ligand-binding domain (from Glu-Lys-His to C terminus; amino acids 98–473) of medaka RORd1 (the same amino acid sequence used in phylogenetic analysis) fused to the DNA binding domain (amino acids 1–161) of the yeast Gal4 transcription factor in the pBIND vector (CheckMate mammalian two-hybrid system, Promega) was made by the following process. The amplicon of the pBIND vector, amplified with T7 and T3 primers, was cloned into a pGEM-T Easy vector (Promega), and the vector was treated with *Not*I. The fragment was subcloned into the *Not*I site of a pcDNA3.1(+) plasmid vector. The resulting plasmid construct was named pcDNA3.1(+)-GAL4DBD vector. The PCR fragment of the RORd1 ligand-binding domain, amplified with the primer set: 5′-GTTGATATCATCTAGCCAGAAACACCGGCA-3′ (forward) and 5′-AATAGGGCCCTCTAGTTAGCCCTCTGTGGA-3′ (reverse), was cloned into a pcDNA3.1(+)-GAL4DBD vector treated with *Xba*I.

A Gal4-regulated reporter vector which contains the luciferase gene under the control of five GAL4 binding sites upstream of a minimal TATA box, named pG5luc, was from the CheckMate mammalian two-hybrid system (Promega). Human embryonic kidney (HEK) 293 cells were seeded in 9 cm dishes before transfection. Both plasmids, i.e., the expression vector for Gal4- RORd1 (1500 ng) and pG5luc (1500 ng), were transfected with X-tremeGENE 9 DNA Transfection Reagent (Roche), following the manufacturer’s instructions. Stably transfected cells were selected with G418 (Nacalai Tesque). Cells derived from the chosen clone were seeded in 96-well luminometer plates with phenol red-free Dulbecco’s modified Eagle’s medium supplemented with 5% dextran-charcoal-treated fetal bovine serum and the test compound (Additional file 1: Table S1); as a preliminary test, each reagent was applied at 10, 1, or 0.1 μM. If significant transactivity was detected in this pre-test, treatment of cells was pursued at four-fold serial dilution from 1 × 10^−5^ M to 6.1 × 10^−10^ M or 3.8 × 10^−11^ M. Cultures were again run; negative control wells were dosed with media containing 0.1% ethanol. After 24 h, the luciferase activities of the cells were measured by a luminescence assay using Steady-Glo Assay System Kit (Promega) according to the manufacturer’s instructions. Concentration-response curves were fitted and the EC_50_ or IC_50_ values were calculated using the sigmoidal fit in GraphPad Prism5 (GraphPad Software, San Diego, CA). The efficacy of each reagent is expressed as a percentage of the reporter induction of EtOH treatment.

### Luciferase reporter assay for medaka *bmal1a* or *cyp26a1*

The entire protein-encoding region (corresponding to amino acids 1–473) and stop codon of the medaka *rord1* (the same amino acid sequence used in phylogenetic analysis) was amplified by PCR and cloned into pcDNA3.1(+) (Invitrogen) at the *Xba*I site by using In-Fusion HD cloning kit (Takara). We named the resulting DNA construct pcDNA-RORd1. A 271-bp fragment of the medaka *bmal1a* promoter region (scaffold212: 822,689–822,960) containing one RORE (*AAAGTGGGTCA*, consensus sequence: WAWNTRGGTCA) and one RORE-like sequence (*T*CC*A*C*GGGTCA*) was isolated by PCR and cloned into the luciferase reporter pGL4.20 vector (Promega) at the *Eco*RV site by using In-Fusion HD cloning kit (Takara). We designated the resulting DNA construct bmal1ap-luc. A 1213-bp fragment of the medaka *cyp26a1* promoter region (chromosome19: 21,523,225–21,524,438) containing two RORE-like sequences (G*A*G*G*A*AGGTCA* and *T*GG*C*GC*GGTCA*) was isolated by PCR and cloned into the luciferase reporter pGL4.20 vector (Promega) at the *Eco*RV site using the In-Fusion HD cloning kit (Takara). We named the resulting DNA construct cyp26a1p-luc. HEK293 cells in 24-well plates were transfected with 500 ng luciferase reporter plasmid (bmal1ap-luc or cyp26a1p-luc) and 50 ng expression vector (pcDNA-RORd1 or pcDNA). Cells were dosed with vehicle (EtOH) or 10^−5^ M 4-hydroxy-ATRA (4OH-ATRA). Cell culture conditions and measurements of luciferase activity were as described above for the pG5luc assays.

## Results

### Phylogenetic analysis

**Fig. 1:**
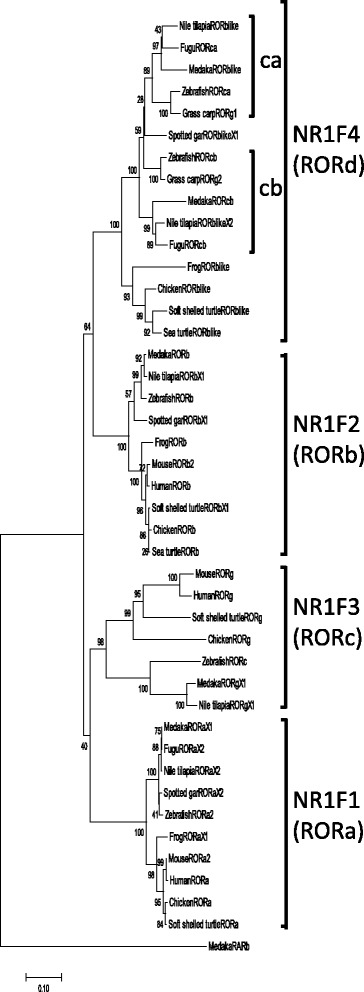
Phylogenetic tree based on the protein sequences of the RORs in vertebrates. Amino acid sequences were aligned and a phylogenetic tree was constructed with the MEGA 7.0.14 software, using the neighbor-joining method. Branch support values estimated using bootstrap tests with 1000 replications are shown next to the branches. A new branch against conventional RORs was described as NR1F4 (RORd)

Using phylogenetic analysis, four groups of *ror*s formed monophyletic clades, supported by bootstrap values >98% (Fig. 1). These clades correspond with *nr1f1* (*rora*)*, nr1f2* (*rorb*)*, nr1f3* (*rorc*) and a new subtype, named *nr1f4* (*rord*). It is conceivable that they are ohnologs. Most non-mammalian species used for our analysis have four *ror* ohnologs. In contrast, mammalian species were not represented in the branch for *nr1f4/rord*, although it is represented among the other three groups, *nr1f1–3*. Other tetrapods (birds, reptiles and amphibians) had one ortholog belonging to the *nr1f4/rord* subtype, while *nr1f4/rord* was clearly subdivided into two distinctive clades, *rord1 and rord2,* in teleost fishes (zebrafish, grass carp, medaka, Nile tilapia, and fugu).

### Spatial distribution for *rord1* mRNA

**Fig. 2:**
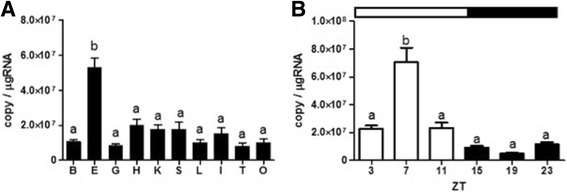
Tissue-specific distribution and time course of expression of medaka *rord1* assessed by quantitative Real-time (RT)-PCR. **a** expression of *rord1* mRNA in various tissues from adult medaka raised under 14 L:10D and sampled at Zeitgeber time ZT7 (7 h after “lights on”). B: brain; E: eye; G: gill; H: heart; K: kidney; L: liver; S: spleen; I: intestine; T: testis; O: ovary. **b** temporal changes in expression of *rord1* in eyes. The sample collection time is indicated as ZT. *White* and *black bars* above each graph represent light and dark periods. Y-axes represent *rord1* expression as copies/μg-total RNA. Data are expressed as means ± standard error of the mean (S.E.M.) (*n* = 6). Different letters on the columns indicate group means that are statistically different when analyzed using one-way ANOVA followed by Tukey’s Multiple Comparison Test (*P* < 0.001)

Significantly higher expression of *rord1* was observed in the eye than in other tissues at ZT7 (Fig. 2a). Employing time-course analysis, *rord1* expression in the eye showed clear diurnal oscillations, increasing during the day to reach a peak at ZT7, before decreasing towards night (Fig. 2b). Since no differences in expression of *rord1* were detected between sexes by *t*-test (*P* values >0.01) (Additional file 2: Figure S1), male and female data were pooled for this analysis.

### Efficacy of typical mammalian ROR ligands for medaka RORd1

**Fig. 3:**
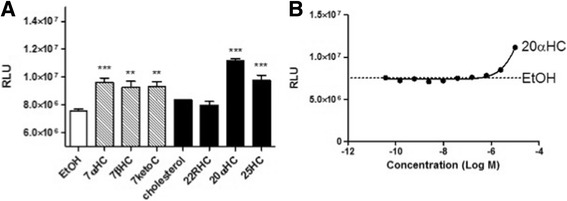
Effects of cholesterols on medaka RORd1. **a** Effects of cholesterols on medaka RORd1 transcriptional activity, assessed by luciferase reporter assay; stably transfected cells were treated with vehicle (control) or 10 μM of cholesterol derivatives. The *white column* is vehicle (ethanol), and the *hatched* and *black columns* represent ROR inverse agonists and agonists in mammals, respectively. Longitudinal axis shows relative luminescence units (RLU). Statistical differences between vehicle and ligands are indicated by asterisks (**: *P* < 0.01; ***: *P* < 0.001). 7α-hydroxycholesterol (7αOHC); 7β-hydroxycholesterol (7βOHC); 7-ketocholesterol (7ketoC); 22(R)-hydroxycholesterol (22RHC); 20α*-*hydroxycholesterol (20αHC); 25-hydroxycholesterol (25HC). **b** Concentration-response curve of 20αHC. Broken line shows the luminescence of ethanol-treated cells. Each point represents the mean of triplicate determinations, and vertical bars represent the S.E.M (**a**, **b**)

In medaka, 20α-OHC was the most potent agonist of RORd1 among the natural cholesterol-related reagents containing well-known ligands for mammalian RORs we tested (Fig. 3a, Table 1). However, the concentration-response curve of 20α-OHC was not sigmoidal and its EC_50_ value could thus not be calculated (Fig. 3b). Other cholesterols yielding significantly increased reporter activity compared to control incubations were 7α-hydroxycholesterol (7α-OHC), 7β-hydroxycholesterol (7β-OHC), 7- ketocholesterol (7 K–C) and 25-hydroxycholesterol (25-OHC); no significant effect was detected in response to treatment with cholesterol or 22R–hydroxycholesterol. The RORdl-dependent transcription was increased slightly in cells treated with 7α-OHC, 7β-OHC, 7 K–C or 25-OHC compared with that in cells treated with EtOH, amounting to 125%, 122%, 127% and 155%, respectively.

**Table 1:** Efficacy of typical ROR ligands in mammals for medaka RORd1

Ligand	Efficacy against EtOH (%)
20α-hydroxycholesterol	1.57 ± 0.06
7α-hydroxycholesterol	1.25 ± 0.01
7β-hydroxycholesterol	1.22 ± 0.00
7-ketocholesterol	1.27 ± 0.06
cholesterol	ND
22R–hydroxycholesterol	ND
25-hydroxycholesterol	1.55 ± 0.11

*ND* not detected

### Screening for endogenous ligands of medaka RORd1

**Fig. 4:**
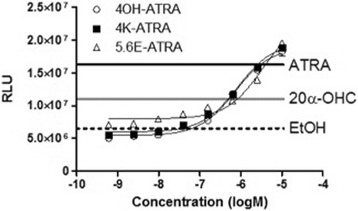
Concentration-response curve of all-*trans* retinoic acid metabolites. Y-axis shows relative luminescent units (RLU). The dashed line represents the average value of controls, the gray solid line signifies the average value of 10^−5^ M 20α-hydroxycholesterol (20α-OHC) and the *black solid line* that of 10^−5^ M all-*trans* retinoic acid (ATRA). Each point represents the mean of triplicate determinations, and *vertical bars* represent the S.E.M. 4OH-ATRA: 4-hydroxy ATRA; 4 K–ATRA: 4-keto ATRA; 5,6E–ATRA: 5,6-epoxy ATRA

To identify potential ligands regulating medaka the transcriptional activity of RORd1, we used a stable cell line expressing a Gal4-RORd1 fusion protein for a reporter gene assay. Fifteen compounds yielded agonistic effects on the medaka RORd1 (Table 2). 4OH-ATRA and 4-keto ATRA (4 K–ATRA), both belonging to the retinoids, showed the highest potency, with EC_50_ values in the order of 100 nM (Fig. 4). The reporter activity of 4OH-ATRA was 3.1-fold higher than that of EtOH, whereas luminescence after addition of 4 K–ATRA was 3.3 times that seen in response to adding EtOH. However, 5,6-epoxy-ATRA (5,6E–ATRA) had lower potency (EC_50_ > 10 μM); administration of 10^−5^ M 5,6E–ATRA showed a 2.8-fold higher reporter-induced luminescence than that observed in controls. Low potency (EC_50_ > 10 μM) was also detected in response to supplementation with other reagents, i.e., three steroids (estradiol, 17-hydroxyprogesterone, and androstenedione), one amino acid derivative (melatonin), and eight retinoids [retinol, retinal, all-*trans* retinoic acid (ATRA), 11-*cis* retinol, 11-*cis* retinal, 11-*cis* retinoic acid, 9-*cis* retinoic acid, 13-*cis* retinoic acid]. ATRA, 11-*cis* retinoic acid, 9-*cis* retinoic acid, and 11-*cis* retinal enhanced luciferase activity in a dose-dependent manner; the fold-induction of these ligands compared to that of EtOH was 2.90, 2.84, 2.39 and 2.37, respectively. By comparison, retinal, 13-*cis* retinoic acid induced approximately twice the luminescence to that of controls. The agonistic activity of estradiol, 17-hydroxyprogesterone and 11-*cis* retinol were higher than that of EtOH (1.73-, 1.62- and 1.67-fold, respectively), whereas androstenedione, melatonin and retinol yielded fold-values of 1.26, 1.37 and 1.29, respectively, lower than the fold-induction observed in response to the mammalian ligand for ROR, 20α-OHC.

**Table 2:** Efficacy and potency of physiologically active substance for medaka RORd1

	Ligand	Efficacy against EtOH (%)	Potency (nM)
Steroid	estradiol	1.73 ± 0.21	NC
	17-hydroxyprogesterone	1.62 ± 0.14	NC
	androstenedione	1.26 ± 0.06	NC
Indoleamine derivative	melatonin	1.37 ± 0.08	NC
Thyroid hormone	triiodothyronine (T3)	−0.154 ± 0.03	NC
Retinoid	all-*trans* retinoic alcohol (VA)	1.29 ± 0.04	NC
	all-*trans* retinoic aldehyde (retinal)	1.95 ± 0.06	NC
	all-*trans* retinoic acid (ATRA)	2.90 ± 0.18	NC
	11-*cis* retinoic alcohol	1.67 ± 0.14	NC
	11-*cis* retinoic aldehyde	2.37 ± 0.14	NC
	11-*cis* retinoic acid	2.84 ﻿±﻿ 0.15	NC
	9-*cis* retinoic acid	2.39 ± 0.08	NC
	13-*cis* retinoic acid	1.98 ± 0.23	NC
	all-*trans* 4-hydroxyretinoic acid	3.07 ± 0.20	539 ± 97.3
	all-*trans* 4-ketoretinoic acid	3.26 ± 0.28	737 ± 47.3
	all-*trans* 5,6-epoxyretinoic acid	2.81 ± 0.31	NC

*NC* not calculable

Not all tested ligands displayed agonistic activity. The amino acid derivative 3,3′,5-triiodo-L-thyronine (T_3_), a thyroid hormone, presented with an inverse agonistic effect on RORd1 in this study (Table 2). The luciferase activity was decreased by 15.4% compared to that in EtOH-supplemented incubations, and the EC_50_ of T_3_ was higher than 10 μM. Other compounds tested had no effect on medaka RORd1.

### Luciferase reporter assay for medaka *bmal1a* or *cyp26a1* and these expression rhythms

**Fig. 5:**
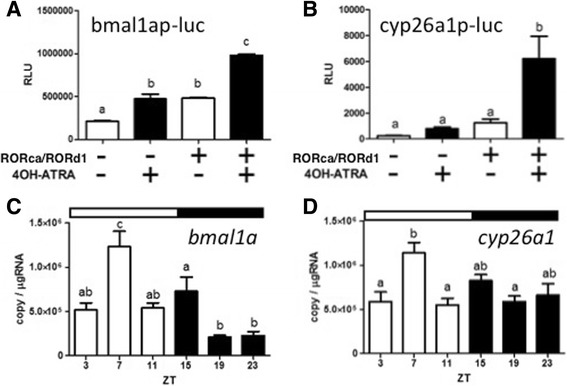
Reporter gene assay using consensus RRE in *bmal1a* or *cyp26a1* promoter of medaka and these expression rhythms. HEK293 cells transfected with (**a**) bmal1ap-luc or (**b**) cyp26a1p-luc and pcDNA-RORd1 or pcDNA were dosed with EtOH (open) or 10^−5^ M 4OH-ATRA (closed). Longitudinal axis shows relative luminescent units (RLU). The temporal change in expression of (**c**) *bmal1a* and (**d**) *cyp26a1* in eyes. The sample collection time is indicated as ZT. *White* and *black bars* above each graph represent light and dark periods. Y-axes represent gene expression as copies/μg-total RNA. Data are expressed as means ± standard error of the mean (S.E.M.) (*n* = 6). Different letters on the columns indicate group means that are statistically different when analyzed using one-way ANOVA followed by Tukey’s Multiple Comparison Test (*P* < 0.001)

We tested a full-length RORd1 in transient transactivation assay to rule out possibility that differences in ROR activity were due to interaction between Gal4 DBD and ROR LBD. Using the bmal1ap-luc reporter, 4OH-ATRA significantly induced luciferase activity relative to EtOH, irrespective of the presence or absence of RORd1 (Fig. 5a). However, with RORd1, luciferase activity was significantly increased compared to without RORd1 for both doses. In contrast, when using the cyp26a1p-luc reporter, a significant increase in luminescence was detected in cells expressed RORd1 and exposed to 4OH-ATRA relative to in other cells, which are exposed to EtOH, not expressed RORd1 and both (Fig. 5b). Time-course analysis indicated that *bmal1a* and *cyp26a1* mRNA expression were similar to that of *rord1* in that a peak showed at ZT7 in the eyes (Fig. 5c, d). Since no differences in expression of rord1 were detected between sexes by t-test (*P* values >0.01) (Additional file 2: Figure S1), male and female data were pooled for this analysis.

## Discussion

The relationships between the different paralogs of RORs in vertebrates have remained unclear, in part due to the abundance of products for this gene family in teleost fish. We therefore set out to first clarify the ROR family relationships. In our phylogenetic analysis, *ror*s were classified into four main groups, a new fourth group being created against the conventional *ror* subtypes, *nr1f1–3*. This additional subtype consists of *rorca, rorcb,* and *rorb*-like homologs. We propose to name this group *rord* or *nr1f4*.

On the basis of the phylogenetic tree, we propose that a proto-ortholog of *ror* in the cephalochordate ancestral genome gave rise to two paralogs, the common ancestor for both *rora* and *rorc* and its counterpart (*rorb* + *rord*), after an initial round of duplication. A total of four orthologs were generated thereafter following a second genome duplication event. Interestingly, orthologs belonging to *nr1f4/rord* were not found in mammalian taxa in any databases. *Nr1f4/rord* thus appears to have been lost in the mammalian lineage during evolution, as these orthologs were present in other tetrapods and in fish. In the latter group, the fourth *ror* subtype gave rise to two daughters, *rord1* and *rord2*, after yet another round of genome duplication; this third duplication is a teleost-specific whole genome-duplication (TWGD) and is believed to have occurred around 320 million years ago [16]. Indeed, the presence of only a single *nr1f4/rord1* ortholog in taxa including chicken, turtle, and the holostean (non-teleost) spotted gar reinforces the notion that *rord1* and *rord2* paralogs appeared as a consequence of TWGD. Although *nr1f1, nr1f2* and *nr1f3* could be expected to also have two paralogs after TWGD, just like *nr1f4*, there was no evidence to support this; two functional NR1F4s are seemingly needed in teleosts, while this subtype appears to be dispensable in mammals.

Among the tissues we examined, the eye showed the highest expression of *rord1* mRNA in adult medaka at ZT7. Expression of *ror* subtypes in various organs, including the eye, has been documented in different species; for example, *Rora* mRNA was detected in moderate quantities in muscle and skin in mouse, but its principal expression site is the brain, particularly the thalamus and Purkinje cell layer of the cerebellum [17]. The *Rorb* gene is highly expressed in the pineal gland, the thalamus and the hypothalamus in the rat. In situ hybridization analysis revealed that the suprachiasmatic nucleus and the inner nuclear layer of the retina in particular are high-expression *Rorb* sites [18]. Similarly, *Rorc* expression has been reported in muscle and the thymus in mammals. In mouse, Rorγt, which is an alternative splicing form of Rorc in the thymus, was shown to be an essential factor for differentiation of naïve CD4^+^T (Th0) cells into Th17 cells, indicating an immunologically relevant function [19].

Unlike their mammalian counterparts, in non-mammalian species only a few studies on localization and function of *ror*s/RORs have been reported. Using whole-mount in situ hybridization analysis, *rora*, *rorb* and *rorc* expression were detected in diencephalon, eyes, pineal gland, pharynx, heart, liver, gut, and somites of the zebrafish embryo [20]. The *rora* and *rorc* were strongly expressed in pituitary, brain and immune-related tissues in grass carp, suggesting that these subtypes are associated with the endocrine and immune systems [21]. In rainbow trout, *rorc* was reportedly highly expressed in muscle, other sites of expression being brain, heart, and skin [22]. In keeping with these findings on *ror*s in fish, medaka *rord1* was also detected in the brain. It is unclear whether *rord1* is expressed universally in the eye, as there are no studies of tissue localization of this subtype in other species. In any case, its conspicuous expression in the eyes suggests that RORd1 may relate to visual functions; indeed, there are two earlier reports that implicate RORa and RORb in the regulation of the expression of opsins, suggestive of a photoreceptor function, in cone cells [23, 24]. Furthermore, RORd1 could be involved in yet-unknown physiological responses characteristic to teleost fish.

*Rord1* mRNA abundance peaked during the middle of the light phase in eyes of medaka in the present study. It is thus tempting to speculate that RORd1 may be involved in synchronizing the circadian clock, or that it plays a role in regulation of rhythmicity. An example of the latter, via RORE, RORs are likely to control the expression level of *bmal1* whose products constitute a core loop of the circadian rhythm [25, 26]. In a promoter assay performed using bmal1a-luc in this study, although 4OH-ATRA stimulated the expression of luciferase without RORd1, significantly higher expression of luciferase was detected in the presence of RORd1 than in the absence of RORd1. This suggest two possibilities: (1) the endogenous 4OH-ATRA receptor binding to bmal1a promoter domain is expressed in HEK293; and (2) the sequence that acts as a RORE resides in the *bmal1a* promoter domain of transfected bmal1a-luc. Thus, RORd1 can enhance *bmal1a* promoter activity. Given the periodic expression of RORd1, this receptor could therefore be involved in the regulation of circadian clocks, at least in the eyes. The finding that the mRNA expression pattern of medaka bmal1a is similar to that of rord1 in eyes supports this notion.

In the present study, we used a heterogeneous system consisting of a mammalian host cell and a Gal4-fusion receptor, which we believe properly represents the transactivational ability of medaka RORd1. It has been suggested that mammalian RORs can function as constitutively active receptors [27], and indeed, constitutive luminescence was observed in the absence of any ligand in the present study. However, forcing the cell line to express the medaka RORd1 resulted in threefold increases in luciferase activity in the presence of a suitable ligand – therefore, RORd1 is likely to act as a bona fide nuclear receptor.

Natural cholesterol-related compounds, which are known ligands for RORs in mammals [27], had varying effects on activity of the medaka RORd1. Among the cholesterol derivatives, 20α-OHC displayed the highest agonist activity for medaka RORd1 in this system. However, the potency of 20α-OHC for medaka RORd1 (EC_50_; > 1 μM) was substantially lower than that for human RORC (EC_50_; 20-40 nM) [5]. Interestingly, 7α-hydroxycholesterol, 7β-hydroxycholesterol and 7-ketocholesterol all showed agonistic activity for medaka RORd1. These findings contrast with their natural inverse agonistic effects on RORa and RORc in mammals, as assessed by decreased transactivation activity [28]. This prompts us to suggest a physiological function for RORd1 in medaka that is distinct from the ROR-mediated role in glucose metabolism in response to binding 7-oxygenated cholesterol in mammals; ROR subtypes other than RORd may perform this function in medaka.

Putative natural agonists for medaka RORd1 were identified in this study as retinoids, rather than cholesterols. A previous study documented that ATRA induced the expression of a Purkinje cell-specific gene ‘synergistically’ with RORa via RAR/RXR [29]. ATRA was further reported as an ‘antagonist’ for RORb [7], in contrast with our results which, for the first time, assign an agonistic effect to a retinoid for ROR. Among all-*trans* retinoids, the more oxidized it was, the higher its efficacy, reflected by ATRA > retinal > retinol. Meanwhile, the stereoisomers 9-*cis* retinoic acid and 13-*cis* retinoic acid affected RORd1 in comparable manner to ATRA, but induced higher reporter activity than did 20α-OHC.

We were particularly interested in the ligand activity of 11-*cis*-retinoid species, as these are generated only in the retina, which is relevant, as *rord1* expression was highest in the eyes. However, 11-*cis*-isomers were no more effective in elevating reporter activity than ATRA. Admittedly, ATRA yielded high transactivation activity mediated by RORd1 but its effective concentration was not calculable (EC_50_ value >1 μM). Therefore, ATRA is too weak to act as a ligand for RORd1 in vivo. In contrast, 4OH-ATRA and 4 K–ATRA yielded EC_50_ values in the sub-μM range, while that of 5,6E–ATRA was not calculable. It is widely held that the predominant route of elimination of ATRA is through its oxidation in the 4-position of the β-ionone ring to generate 4OH-RA [30, 31]. Here, we show that ATRA metabolites may serve as bona fide ligands for RORd1, reflecting their high potency for this receptor.

Retinoic acid plays an important role as a morphogen during embryonic development [32]. However, catabolism of retinoic acid is also essential. The concentration of retinoic acid is regulated by Cyp26, a hydroxylase that degrades ATRA into 4OH-ATRA [33–35], highlighting the importance of this enzyme for proper development [36]. A recent study revealed the existence of a retinoic acid concentration gradient in the zebrafish embryo by 14 h after fertilization; retinoic acid levels were low in the head and tail, which was due to local expression of *cyp26*s (*cyp26a1*, *cyp26b1* and *cyp26c1*) responsible for retinoic acid conversion into its metabolites [37]. In one previous study, *rora, rorb* and *rorc* message RNAs were detected in the head and other areas of the zebrafish embryo [19]; these ROR subtypes conceivably receive ATRA metabolites, as shown for medaka RORd1. It appears likely that RORs function as a key regulator in a positive feedback loop resulting in the rapid degradation of ATRA, as RORs serve as sensors for ATRA metabolites and upregulate the expression of these enzymes for catabolism. Further studies of the distribution of RORd1 in the medaka embryo are necessary to define the role of this receptor during development. Aside from its role in development, RORd1 may also be required for hematopoiesis or immunity, as our results indicate that this subtype is expressed at around 10^7^ copies/μg-RNA in a suite of tissues in adult medaka. Our finding, obtained by qPCR, that *cyp26a1* mRNA levels increase at nearly the same time with *rord1* expression suggests that medaka RORd1 may primarily regulate the transactivation of *cyp26a1* gene, and consequently induce ATRA metabolism, at least in the eye, at ZT7. The results of our luciferase reporter assay using cyp26a1p-luc support this possibility.

A promoter assay using cyp26a1p-luc demonstrated that one of the four ROR subtypes, RORd1, is activated by 4-oxygenated ATRA’s metabolites. It is unclear whether these metabolites also act as agonists for other ROR subtypes, prompting the need for further study to compare and define ligand selectivity and physiological function. In any case, a suit of endogenous compounds, including 17-hydroxyprogesterone, melatonin, estradiol, androstenedione, and T_3_, stimulated or inhibited the transcriptional activity in HEK293 cells expressing Gal4-RORd1 LBD. Despite modulation of transcriptional activity, however, we suggest that these compounds do not normally interact with RORd1 in vivo, given the significantly lower ED_50_ for RORd1 compared to that of ATRA metabolites.

## Conclusions

We have characterized a new member of the ROR family, *rord1*, in medaka by analyzing molecular phylogeny, tissue-dependent expression patterns, pharmacological profile, and transactivational activity of the promoters of putative target genes. Phylogenetic tree analysis showed that *rord1* is a novel *ror* subtype that differs from the previously reported subtypes: *rora, rorb* and *rorc*. Two paralogs, *rord1* and *rord2*, are thought to have diverged after the TWGD. *Rord1* mRNA was highly abundant in the eyes of medaka during the light period, suggesting that *rord1* expression is regulated by circadian rhythm. Using a stable cell line expressing a Gal4DBD-RORd1 fusion protein, two ATRA metabolites, 4OH-ATRA and 4 K–ATRA, were found as endogenous candidate ligands for RORd1. Furthermore, we demonstrated that 4-oxygenated ATRA metabolites have the potential to activate the promoter of *cyp26a1*, the metabolic enzyme of ATRA, through RORd1. Our results support the notion that RORd1 is involved in the metabolism of ATRA in medaka.

## Additional files

Additional file 1: Table S1. Tested compounds. (XLSX 11 kb)

Additional file 2: Figure S1. No sex difference was shown in tissue-specific distribution and time course of expression of medaka genes assessed by quantitative Real-time (RT)-PCR (*A*) Expression of *rord1* mRNA in various tissues from adult medaka raised under 14L10D and sampled at Zeitgeber time ZT7 (7 h after “lights on”). B: brain; E: eye; G: gill; H: heart; K: kidney; L: liver; S: spleen; I: intestine; T: testis; O: ovary. Time course of expression of medaka *rord1* (*B*), *bmal1a* (*C*), and *cyp26a1* (*D*). The sample collection time is indicated as ZT. White and black bars above each graph represent light and dark periods. Y-axes represent gene expression as copies/ μg-total RNA. Male is shown in black column, and female is shown in white column. Data are expressed as means ± standard error of the mean (S.E.M.) (*n* = 3). Different letters on the columns indicate group means that are statistically different when analyzed using one-way ANOVA followed by Tukey’s Multiple Comparison Test (*P* < 0.001). (JPEG 67 kb)
