# Supplementary figures and images for: A fourth subtype of retinoic acid receptor-related orphan receptors is activated by oxidized all-trans retinoic acid in medaka (Oryzias latipes)
Source: Zoological Lett. 2017 Aug 11;3:11. doi: 10.1186/s40851-017-0074-7 (PMC5553892; doi:10.1186/s40851-017-0074-7)

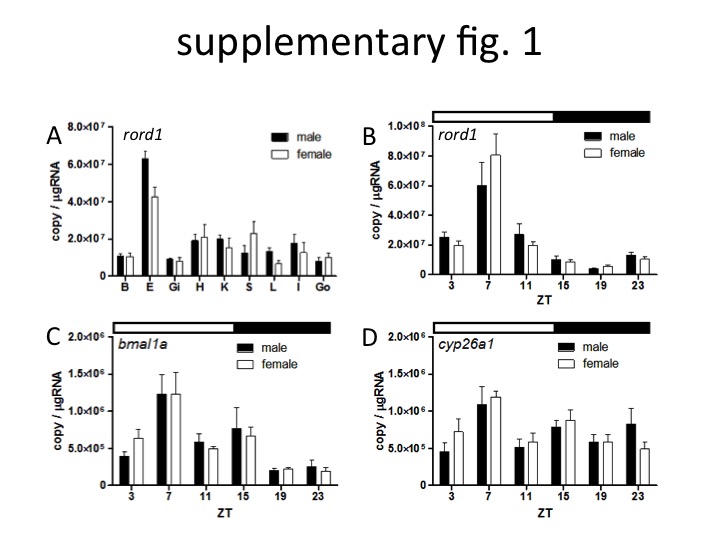

Supplement: Supplementary file 2 — No sex difference was shown in tissue-specific distribution and time course of expression of medaka genes assessed by quantitative Real-time (RT)-PCR (A) Expression of rord1 mRNA in various tissues from adult medaka raised under 14L10D and sampled at Zeitgeber time ZT7 (7 h after “lights on”). B: brain; E: eye; G: gill; H: heart; K: kidney; L: liver; S: spleen; I: intestine; T: testis; O: ovary. Time course of expression of medaka rord1 (B), bmal1a (C), and cyp26a1 (D). The sample collection time is indicated as ZT. White and black bars above each graph represent light and dark periods. Y-axes represent gene expression as copies/ μg-total RNA. Male is shown in black column, and female is shown in white column. Data are expressed as means ± standard error of the mean (S.E.M.) (n = 3). Different letters on the columns indicate group means that are statistically different when analyzed using one-way ANOVA followed by Tukey’s Multiple Comparison Test (P < 0.001). (JPEG 67 kb) [file 40851_2017_74_MOESM2_ESM.jpg]
